# Changes in T and B cell subsets in end stage renal disease patients before and after kidney transplantation

**DOI:** 10.1186/s12979-021-00254-9

**Published:** 2021-11-08

**Authors:** Lei Wang, Christien Rondaan, Anoek A. E. de Joode, Elisabeth Raveling-Eelsing, Nicolaas A. Bos, Johanna Westra

**Affiliations:** 1 Department of Rheumatology and Clinical Immunology, University Medical Center Groningen and University of Groningen, Hanzeplein 1, P.O. Box 30.001, 9700 RB Groningen, NL The Netherlands; 2 Department of Medical Microbiology and Infection Prevention, University Medical Center Groningen, University of Groningen, Groningen, The Netherlands; 3 Department of Internal Medicine, Division of Nephrology, University Medical Center Groningen, University of Groningen, Groningen, The Netherlands

**Keywords:** Kidney transplant, Ageing, CMV, Senescent T cells, Age associated B cells

## Abstract

**Background:**

The incidence of kidney transplantation performed in elderly patients has increased steadily recently. Higher risk of infection and mortality, but lower rate of rejection, are reported in older kidney transplant patients. This study aims to analyze the effect of transplantation on aging of T and B cells in kidney transplant patients, with the emphasis on age and Cytomegalovirus (CMV) latency.

**Results:**

We included 36 patients before and after (median 2.7 years) kidney transplantation and 27 age- and sex-matched healthy controls (HC). T and B cell subsets were measured by flow cytometry, with a focus on aged T cells (CD28-), and age associated B cells (ABCs, CD19 + CD21-CD11c+). Three years after transplantation a significant increase of total T cells among the lymphocytes was found compared to pre-transplantation and HC. Among the T cells CD4+ cells were decreased, especially naïve CD4+ cells and regulatory T cells. Total CD8+ cell proportions were increased, and proportions of naïve CD8+ cells were significantly decreased after transplantation, while CD8+ effector memory T cells re-expressing CD45RA were increased. CD28− T cells were significantly higher compared to HC after transplantation, especially in CMV seropositive patients. B cells were significantly decreased, while among B cells memory B cells and especially ABCs were increased after transplantation.

**Conclusions:**

After transplantation T and B cell subsets change towards more terminally differentiated memory cells compared to age-matched HC. Proportions of aged T cells and ABCs were associated with CMV serostatus.

**Supplementary Information:**

The online version contains supplementary material available at 10.1186/s12979-021-00254-9.

## Background

Kidney transplantation is a final treatment for end-stage renal disease (ESRD) patients compared to dialysis, with lower costs, better quality of life and much higher survival rate [1]. The incidence of kidney transplantation performed in elderly patients shows a continuous growth in recent years. In the US, kidney transplantation incidence was 17.2% in 2012 in patients over 65 years old, and increased four times compared to the incidence in 1990. Similar trends are also seen in Europe [2]. Although age is no longer contraindicative for transplantation, older kidney transplant patients are still at higher risk of morbidity and mortality. Major complications causing mortalities, including infection, cardiovascular disease, and malignancies are partly exacerbated by immunosuppressants. Notably, a lower risk of acute rejection was observed in elderly patients, which was believed to be due to their impaired immune reactivity, or so-called “immunosenescence” [3].

Immunosenescence is related to thymus involution, showing alteration of the numbers and proportions of the lymphocyte populations. In elderly people, a weak adaptive immune response, vulnerability to resist infections, a higher production of autoantibodies and more inflammatory responses were reported during immunosenescence [4]. Ageing of the immune system may affect all immune compartments, especially T cells. With ageing, the numbers of CD4+ T helper cells and CD8+ cytotoxic T cells decrease, together with less naïve lymphocytes and accumulation of memory lymphocytes [5]. This process is usually accompanied by loss of expression of co-stimulatory surface molecules, such as CD28. CD28 is a key co-stimulatory receptor for T cell activation, proliferation, and survival [6]. Studies showed that CD28− T cells are resistant to apoptosis and decline of CD28 expression on both CD4+ and CD8+ T cells is related to increasing age and T cell senescence. The accumulation of CD28− T cells with age has been shown to be associated with functional impairment, reduced immune responses to vaccines and impaired proliferative ability in response to antigenic and mitotic stimuli [6–8]. The number of CD28− T cells was also found to be positively related to latent Cytomegalovirus (CMV) infection. Primary infection of CMV mainly occurs early in life, after which CMV establishes a lifelong latency. Reactivation of CMV periodically happens and continuous asymptomatic challenges by CMV result in accelerated immunosenescence [9].

The B cell compartment also shows intrinsic alterations in the elderly, including impaired B cell development in the bone marrow, leading to a decrease both in the number and percentage of total CD19+ B cells and reduced antibody production ability [10]. In 2011, a unique B cell subset termed age-associated B cells (ABCs) was identified by Hao et al. and Rubstov et al. independently, with a characteristic profile of CD11c + CD11b + CD21 − CD23− expression that accumulates both in aged mice as well as in aged humans [11, 12]. This increase has also been seen in people with autoimmune diseases. In addition, it has been suggested that ABCs are not self-renewing cells. ABCs displace follicular B cells with advancing age while the sum of ABCs and follicular B cells remains relatively stable, which would eventually lead to a B cell pool with ABCs and immunosenescent features [13]. Since most studies about the function of ABCs were done in mouse models, still more research is needed on how ABCs interact with other T/B cell subsets in aged humans.

In this study, we investigated the phenotype of T and B cells in kidney transplant patients before and around 3 years after transplantation, compared with healthy age−/sex- matched controls. We specially focused on aged T cells (CD28−) and ABCs (CD21 − CD11c+) in relation to age and CMV latency of the transplant patients.

## Results

### Participant characteristics

In total, 36 kidney transplant patients and 27 healthy controls (HC) were enrolled in this study (Table 1). The main cause of renal failure was glomerulonephritis (41.7%), especially IgA nephropathy (25%). All the pre-transplantation (pre-Tx) blood samples were taken right before kidney transplantation except in one patient which was about 3 months before transplantation. The average follow-up time post-transplantation (post-Tx) was 2.7 years, with a range of 2.2 to 3.1 years. There was no significant difference in age and gender between transplant patients and HC. Most of the patients (80.6%) received tacrolimus, mycophenolate mofetil and prednisone as maintenance immunosuppressant therapy.

**Table 1:** Characteristics of healthy controls and transplant patients

	Healthy controls (*N* = 27)	Transplant patients (*N* = 36)
Age, median (range) years	52.3 (20.2–64.0)	51.3 (25.6–68.2)^a^
Female gender, no. (%)	14 (51.9)	20 (55.6)
CMV serostatus, no. (%)^b^		
+	10 (37.0)	21 (58.3)
-	17 (63.0)	9 (25.0)
Donor (D)/Recipient (R) CMV serostatus, no. (%)		
D+/R+ & D−/R+		21 (58.3)
D+/R-		3 (8.3)
D−/R-		12 (33.3)
Temporary (val) ganciclovir, no. (%)^c^		8 (22.2)
Cause of renal failure, no. (%)		
Glomerulonephritis		15 (41.7)
IgA nephropathy		9 (25.0)
MPGN		2 (5.6)
FSGS		2 (5.6)
Anti-GBM		1 (2.8)
SLE		1 (2.8)
Vascular		3 (8.3)
Diabetic nephropathy		3 (8.3)
Genetic		4 (11.1)
Urologic		3 (8.3)
Congenital		1 (2.8)
Chronic TIN		1 (2.8)
Unknown		6 (16.7)
Immunosuppression		
Tac/MMF/Pred		29 (80.6)
Cyc/MMF/Pred		4 (11.1)
Tac/MMF/Pred to Cyc/MMF/Pred^d^		2 (5.6)
Everolimus/MMF/Pred		1 (2.8)
History of > 1 Tx, no. (%)		6 (16.7)
RRT pre-Tx, no. (None/HD/PD)		9/16/11
Pre-Tx lymphocytes, × 10^9^ cells/l, median (IQR)^e^		1.7 (1.2–2.2)
Rejection		6 (16.7)
Infection, any/bacterial/viral		28/16/21

Abbreviations: *Tx* transplantation, *CMV* Cytomegalovirus, *MPGN* membranoproliferative glomerulonephritis, *FSGS* focal segmental glomerulosclerosis, *GBM* glomerular basement membrane, *SLE* systemic lupus erythematosus, *TIN* tubulointerstitial nephritis, *Tac* tacrolimus, *Cyc* cyclosporine, *MMF* mycophenolate mofetil, *Pred* prednisolon, *RRT* renal replacement therapy, *HD* haemodialysis, *PD* peritoneal dialysis, *IQR* interquartile range. ^a^Age at Tx. ^b^patients with consistent CMV serostatus. ^c^CMV-load of patients was evaluated after transplantation and (val) ganciclovir was given if positive load was found. Treatments stopped when CMV-load < 100 copies/ml for two times. ^d^2 patients switched from Tac to Cyc owing to side effects. ^e^Data of lymphocytes counts were available in 33 patients

Twenty-one patients were CMV seropositive at time of transplantation, which was higher than in HC (58.3% vs 37%). A total of 8 patients received temporary (val) ganciclovir treatment. Among them, 5 patients were already CMV seropositive at time of transplantation. The other 3 patients experienced a primary CMV infection in the post-transplantat period. Another 3 patients CMV-seroconverted in the post-transplant period without reported antiviral treatment. Because we aimed to analyze the effect of stable CMV serostatus on the immune system, the six patients with converted CMV serostatus were excluded from analysis for determining the effect on CMV serostatus on frequency of aged B and T cells. After transplantation, most patients did not experience rejection (83.3%) but many patients experienced some bacterial or viral infection or both (77.8%).

### The effect of kidney transplantation on T cell subsets

We investigated the relative percentage of T cell subsets among CD3+ T cells in kidney transplant recipients pre- and post-Tx, compared with HC (Table 2). Overall, no difference was found in the percentage of total T cells among all lymphocytes and in the T cell subsets between pre-Tx and HC.

**Table 2:** Frequency of T cell subsets in the healthy controls and kidney transplant patients pre- and post-transplantation

	pre-Tx (*N* = 36)	post-Tx (*N* = 36)	HC (*N* = 27)	*p* value		
				pre-Tx vs post-Tx	HC vs pre-Tx	HC vs post-Tx
CD3+/Lymphocytes (%)	65.7 (59.5–74.6)	74.7 (67.3–81.5)	69.7 (67.0–74.0)	0.001	ns	0.022
CD4+/CD3+ (%)	58.9 (48.6–72.9)	52.0 (41.0–67.7)	62.1 (49.3–71.5)	0.007	ns	0.042
Naïve CD4+/CD4+ (%)	34.0 (18.8–47.8)	22.3 (13.2–36.6)	36.8 (27.8–47.8)	0.002	ns	0.005
CM CD4+/CD4+ (%)	28.0 (21.5–33.8)	25.3 (19.8–32.8)	28.7 (22.9–34.3)	ns	ns	ns
EM CD4+/CD4+ (%)	33.9 (19.3–43.0)	39.6 (26.7–51.0)	28.9 (21.3–36.9)	0.002	ns	0.004
TEMRA CD4+/CD4+ (%)	4.1 (3.0–6.3)	4.2 (3.3–7.4)	4.3 (3.1–5.9)	ns	ns	ns
Tfh CD4+/CD4 + CXCR5+ (%)	6.2 (4.0–8.9)	6.0 (3.8–10.0)	5.8 (4.0–13.0)	ns	ns	ns
Treg CD4+/CD4+ (%)	5.1 (3.8–6.6)	3.8(2.9–5.6)	5.7 (4.7–6.5)	0.005	ns	0.003
CD4 + CD28−/CD4+ (%)	2.6 (1.1–7.3)	3.6 (1.3–9.3)	1.5 (0.6–3.1)	ns	ns	0.027
Naïve CD4+/CM CD4+ ratio	1.1 (0.6–1.8)	0.8 (0.6–1.7)	1.3 (0.8–1.9)	0.036	ns	ns
Naïve CD4+/EM CD4+ ratio	1.0 (0.5–2.4)	0.6 (0.3–1.3)	1.1 (0.8–2.1)	< 0.001	ns	0.003
CD8+/CD3+ (%)	25.8 (17.4–34.6)	31.4 (21.7–45.1)	26.1 (21.3–32.2)	0.003	ns	ns
Naïve CD8+/CD8+ (%)	23.0 (16.7–53.1)	19.3 (8.6–37.9)	31.7 (18.4–53.8)	< 0.001	ns	0.019
CM CD8+/CD8+ (%)	3.4 (2.4–6.5)	2.3 (1.5–4.0)	3.4 (2.6–5.2)	< 0.001	ns	0.010
EM CD8+/CD8+ (%)	34.1 (24.0–43.8)	33.6 (22.0–47.3)	31.2 (25.3–49.8)	ns	ns	ns
TEMRA CD8+/CD8+ (%)	24.6 (17.5–34.7)	36.5 (28.2–48.9)	19.7 (13.9–34.3)	< 0.001	ns	0.002
CD8 + CD28−/CD8+ (%)	39.3 (19.3–52.8)	43.0 (21.8–65.6)	26.8 (16.7–42.8)	ns	ns	0.010
CD4+/CD8+ ratio	2.3 (1.5–3.9)	1.6 (0.9–2.8)	2.3 (1.3–3.3)	0.002	ns	0.049

Abbreviations: *HC* healthy controls,*Tx* transplantation, *CM* central memory, *EM* effector memory, *TEMRA* effector memory T cells re-expresses CD45RA, *Tfh* T follicular helper cells, *Treg* regulatory T-cell. Median (interquartile range) of the percentage is shown. *p*-value more than 0.05 was regarded as non-statistically significant (ns)

After transplantation, many changes occurred in the composition of lymphocyte subsets. The percentage of CD4+ cells significantly decreased while CD8+ cells percentages increased post-Tx, which was reflected in the reduced CD4+/CD8+ ratio.

Among CD4+ T cells, the main change was seen in the percentage of naïve and effector memory (EM) cells. The percentage of naïve cells significantly decreased from 34.0 to 22.3% (*p* = 0.002) while EM increased from 33.9 to 39.6% (*p* = 0.002). Besides, in post-Tx samples a decreased percentage of regulatory T-cells (Treg) was found, which was significantly lower compared both to pre-Tx samples and to HC. Similar to CD4+ T cells, CD8+ naïve cells also decreased after transplantation. In contrast to the low percentage of CD4+ effector memory T cells re-expressing CD45RA (TEMRA) in transplant patients and HC, CD8+ TEMRA cells were at a higher level (median 24.6%) before transplantation in patients compared to HC and increased even further after transplantation (median 36.5%, *p* < 0.001).

Finally, we observed a higher percentage of CD4 + CD28− and CD8 + CD28− in patients post-Tx, which were significantly higher (*p* = 0.027 and *p* = 0.010 respectively) as compared to HC.

### The effect of age and CMV latency on CD4+ T cells and CD4 + CD28− T cells

**Fig. 1:**
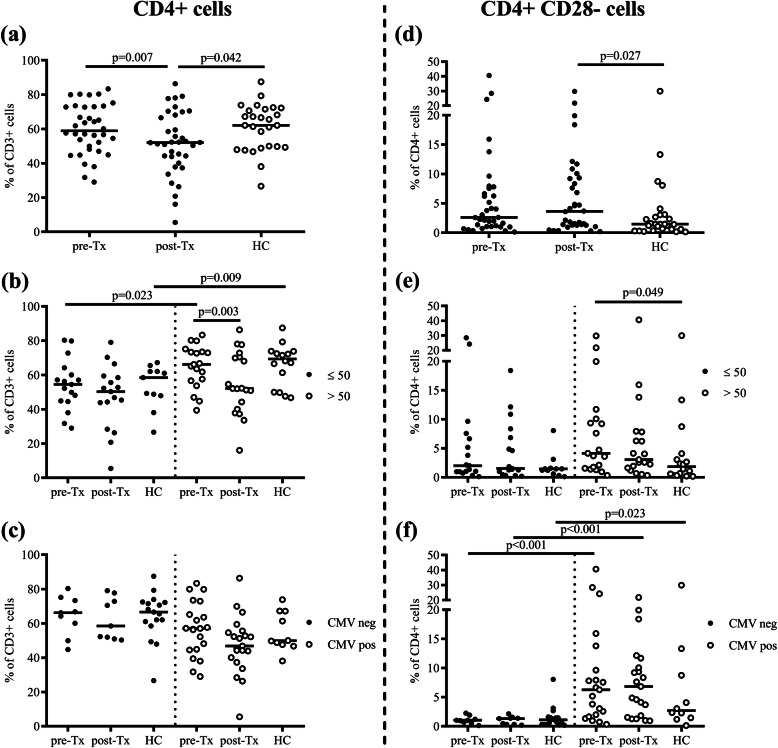
Frequency of CD4+ cells and CD4 + CD28− cells. Frequency of CD4+ cells within CD3+ cells in all subjects (**a**) and subgroups by their age (≤50 or > 50 years old) (**b**) or CMV serostatus (**c**). Frequency of CD4 + CD28− cells within CD4+ cells in all subjects (**d**) and subgroups by their age (≤50 or > 50 years old) (**e**) or CMV serostatus (**f**). Lines show the median. Patients with converted CMV serostatus during study period were not included in (**c**) and (**f**). HC, healthy controls; Tx, transplantation

To study the effect of ageing and CMV latent infection on CD4+ T cells and aged (CD28−) CD4+ T cells, different subgroups according to the age and CMV serostatus were made (Fig. 1). Patients were divided in groups below (*n* = 17) and above (*n* = 19) 50 years of age at transplantation (median age was 51 years).

As shown above, the percentage of CD4+ T cells decreased after transplantation (Fig. 1a). In patients over 50 years of age the decrease of CD4+ T cells was significantly lower (*p* = 0.003) compared to younger patients, while elderly HC had a significantly higher percentage of CD4+ T cells compared to younger HC (Fig. 1b). Besides, a significantly decrease of the percentage of naïve CD4+ T cells and increase of CD4+ EM T cells after transplantation was seen mainly in patients above 50 years of age (Supplementary Fig. 1).

The percentage of CD4 + CD28− T cells was higher in post-Tx patients compared to HC (Fig. 1d), but no difference was seen between the two age groups (Fig. 1e). Of note, the percentage of aged CD4+ T cells was significantly higher in CMV seropositive kidney transplant patients (pre- and post-Tx) as well as in HC, compared to CMV seronegative persons (Fig. 1f).

### The effect of age and CMV latency on CD8+ T cells and CD8 + CD28− T cells

**Fig. 2:**
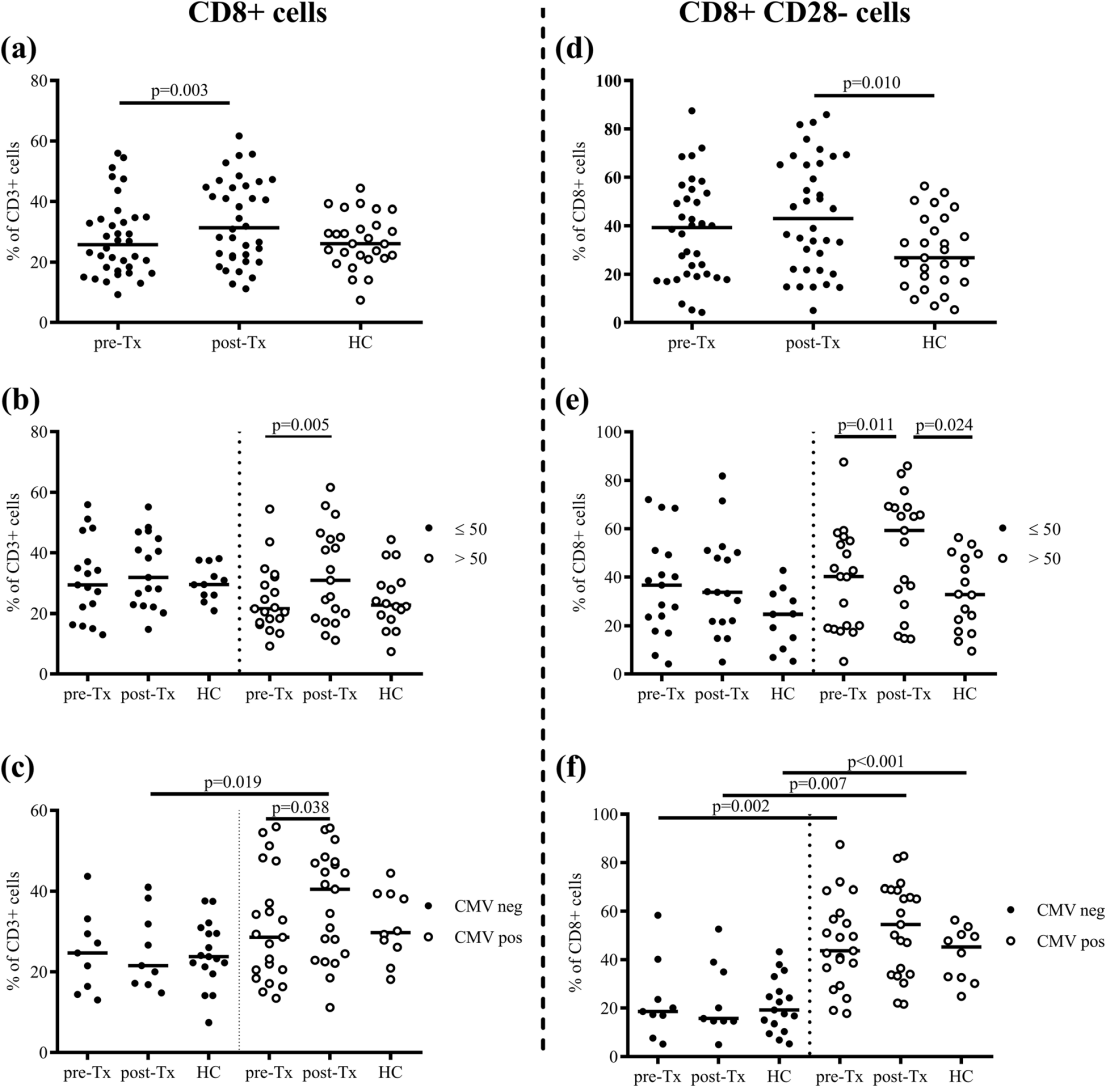
Frequency of CD8+ cells and CD8 + CD28− cells. Frequency of CD8+ cells within CD3+ cells in all subjects (**a**) and subgroups by their age (≤50 or > 50 years old) (**b**) or CMV serostatus (**c**). Frequency of CD8 + CD28− cells within CD8+ cells in all subjects (**d**) and subgroups by their age (≤50 or > 50 years old) (**e**) or CMV serostatus (**f**). Lines show the median. Patients with converted CMV serostatus during study period were not included in (**c**) and (**f**). HC, healthy controls; Tx, transplantation

The effects of ageing and CMV serostatus on total CD8+ T cells and CD8 + CD28− senescent T cells were studied in the previously mentioned groups (Fig. 2).

CD8+ T cell frequencies significantly increased after transplantation (Fig. 2a). This was mainly seen in patients older than 50 years (Fig. 2b) and strongest in CMV seropositive patients (Fig. 2c). For the CD8 + CD28− T cells, although no expansion was seen after transplantation in the total group (Fig. 2d), in older patients higher percentages of CD8 + CD28− T cells after transplantation were found compared to pre-Tx (*p* = 0.011) and older HC (*p* = 0.024) (Fig. 2e). More importantly, CD8 + CD28− T cell frequencies were significantly higher in CMV seropositive subjects (Fig. 2f), likewise CD4 + CD28− T cells in these subgroups. In addition, a higher percentage of CD8+ TEMRA cells was observed in all CMV seropositive individuals (pre−/post-Tx and HC) compared to CMV seronegative individuals (Supplementary Fig. 2).

### The effect of kidney transplantation on B cell subsets

Next, the B cell subsets in kidney transplant patients and HC were analyzed (Table 3). The percentage of naïve and transitional B cells significantly decreased while memory B cells significantly increased after transplantation. No significant difference in most of the B cell subsets was seen, only higher percentages of switched memory B cells and lower percentages of non-switched memory B cells in transplant patients compared to HC. With regard to ABCs, a significantly increased percentage was seen in patients post-Tx compared to pre-Tx (*p* = 0.003).

**Table 3:** Frequency of B cell subsets in the healthy controls and kidney transplant patients pre- and post-transplantation

	pre-Tx (*N* = 36)	post-Tx (*N* = 36)	HC (*N* = 27)	*p* value		
				pre-Tx vs post-Tx	HC vs pre-Tx	HC vs post-Tx
CD19+/Lymphocytes (%)	6.2 (3.6–9.5)	2.9 (2.0–4.7)	5.9 (4.7–8.6)	< 0.001	ns	< 0.001
Naive B cells/CD19+ (%)	64.4 (51.8–73.2)	61.9 (44.5–71.5)	63.9 (52.5–68.5)	0.032	ns	ns
Transitional B cells/CD19+ (%)	2.0 (0.3–3.5)	0.7 (0.4–2.1)	1.2 (0.5–3.3)	0.020	ns	ns
PB/PC B cells /CD19+ (%)	0.4 (0.3–1.3)	0.5 (0.2–0.9)	0.6 (0.4–1.1)	ns	ns	ns
Memory B cells /CD19+ (%)	26.7 (20.2–44.6)	33.4 (26.6–51.6)	32.3 (24.9–43.2)	0.011	ns	ns
Switched Memory B cells /Memory B cells (%)	50.6 (40.6–57.6)	47.3 (36.6–58.2)	38.5 (32.7–43.5)	ns	< 0.001	0.004
Non-Switched Memory B cells/Memory B cells (%)	38.5 (26.2–47.7)	39.9 (26.2–51.7)	52.2 (45.9–56.0)	ns	< 0.001	< 0.001
IgM Only Memory B cells/Memory B cells (%)	2.6 (1.7–4.6)	2.3 (1.6–3.8)	2.6 (1.5–4.0)	ns	ns	ns
ABCs/CD19+ (%)	5.3 (3.5–8.1)	8.0 (5.3–11.0)	6.0 (5.3–7.2)	0.003	ns	ns

Abbreviations: *HC* healthy controls, *Tx* transplantation, *PB/PC* plasma blast/plasma cell, *ABCs* age associated B cells. Median (interquartile range) of the percentage is shown. *p*-value more than 0.05 was regarded as non-statistically significant (ns)

### The effect of age and CMV latency on total B cells and ABCs

**Fig. 3:**
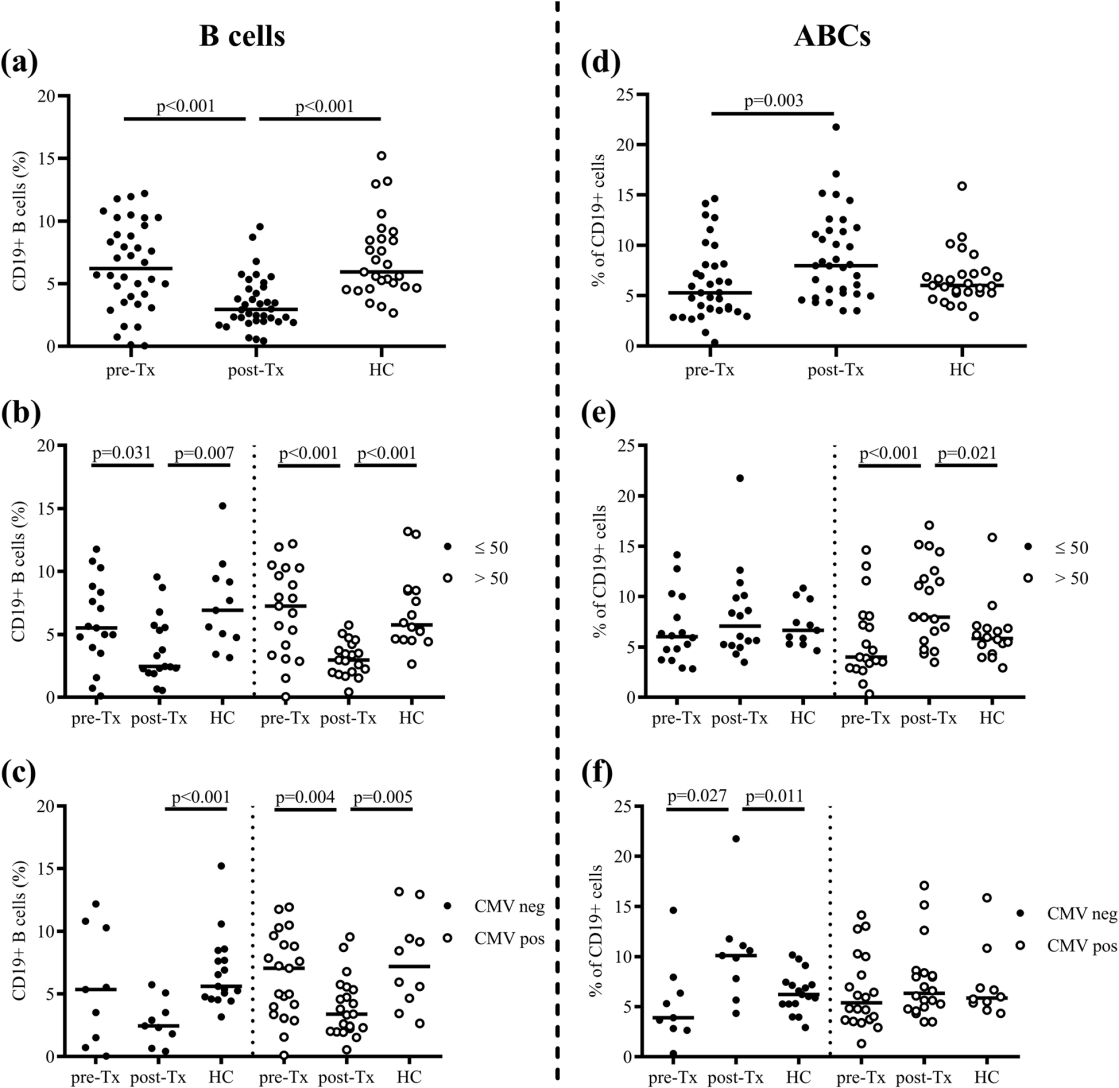
Frequency of B cells and age associated B cells (ABCs). Frequency of CD19+ B cells among lymphocytes in all subjects (**a**) and subgroups by their age (≤50 or > 50 years old) (**b**) or CMV serostatus (**c**). Frequency of ABCs within CD19+ cells in all subjects (**d**) and subgroups by their age (≤50 or > 50 years old) (**e**) or CMV serostatus (**f**). Lines show the median. Patients with converted CMV serostatus during study period were not included in (**c**) and (**f**). HC, healthy controls; Tx, transplantation

To study the relation between age, CMV latency and frequency of total B cells and ABCs, subjects were divided into subgroups by their age and CMV serostatus as above (Fig. 3).

After transplantation, the percentage of B cells was significantly lower than pre-transplantation (*p* < 0.001) also compared to HC (*p* < 0.001) (Fig. 3a). However, there was no difference between young and older patients (Fig. 3b), and between CMV seronegative and CMV seropositive individuals (Fig. 3c). Frequencies of ABCs significantly increased after transplantation (Fig. 3d) especially in older patients (Fig. 3e). Interestingly, we observed clearly elevated ABCs frequencies in CMV seronegative patients after transplantation, which were also higher than in CMV seronegative HC (Fig. 3f). In our study 84% of the patients > 50 years old experienced an infection during follow-up whereas the infection rate was 71% in younger patients. Patients who experienced an infection had significant higher frequencies of EM CD4+ and TEMRA CD8+ cells, and also significantly (*p* = 0.003) increased percentages of ABCs after transplantation, which was not seen in patients who did not (Supplementary Fig. 3).

## Discussion

In this study, we performed flow cytometry to investigate the relative distribution of T and B lymphocytes, especially senescent CD4+/CD8+ T cells and ABCs, in patients before and after kidney transplantation, compared to healthy individuals. Differences in frequency of T and B cell compartments between HC and patients were mainly found after transplantation.

Since the effects of ageing on T cell compartments are considered to be related to transplant outcomes and even transplantation tolerance, great efforts have been devoted to investigate the relationship between immunosenescence and dynamic changes of T cell compositions [7]. Although it has been described that premature ageing of the immune system is seen in patients with ESRD [14], frequencies of T cell subsets in our patient group were not different when comparing patients pre-Tx with HC.

Elderly patients are more vulnerable to infections after transplantation which could be due to an impaired T cell repertoire caused by immunosenescence [7]. In our study 84% of the patients > 50 years old experienced an infection during follow-up whereas the infection rate was 71% in younger patients. In a study investigating T cell subsets in older and younger patients 3 months after kidney transplantation, decreased frequencies of naïve CD4+/CD8+ cells and elevated frequencies of differentiated cells were seen in older patients [15]. In our study, around 3 years after transplantation, significantly decreased frequencies of both naïve CD4+ and naïve CD8+ cell were found while EM CD4+ and TEMRA CD8+ cell frequencies were significantly increased especially in patients over 50 years old (Supplementary Fig. 1, 2). The decrease in ratio of circulating naïve to memory T cells may partly explain the vulnerability to infections in elderly patients. As seen in Supplementary Fig. 3, patients who experienced an infection during the study period had significant higher frequencies of EM CD4+ and TEMRA CD8+ cells. Our finding about the decreased frequency of Treg after transplantation was not in line with a previous study which suggested that CD4 + CD25highFOXP3+ Tregs frequencies remain stable during maintenance immunosuppression after kidney transplantation [16]. Currently, the impact of ageing and transplantation on Tregs remains controversial and this could due to differences in time frame, population or medication usage in these studies [17–19].

Studies have shown that a persistent antigen challenge leads to T cells proliferation and differentiation, and eventually causes the loss of CD28 during cellular senescence. CD28− T cells are considered as terminally differentiated senescent cells, with shortened telomeres and great ability of cytotoxicity [20]. CMV seropositivity was found to be related to accumulation of CD28− T cells. Shabir et al. reported a higher frequency of CD4 + CD28− T cells in CMV seropositive recipients when compared to CMV seronegative recipients 1 year after kidney transplantation. Similar results were found for CD8 + CD28− T cells in other studies [21–24]. This is in agreement with our observation of significantly greater percentages of CD4 + CD28− and CD8 + CD28− T cells in CMV seropositive HC and also in ESRD patients pre- and post-Tx. Interestingly, there was no difference in CD28− T cell frequency in patients pre-Tx compared to post-Tx, and also not between young and old patients. Our data suggest that CD28− T cell expansion in transplant patients is mainly driven by CMV latency instead of ageing or transplantation. In addition, our results show that although the percentage of CD8+ TEMRA and CD8 + CD28− cells was comparable in all subjects, significantly higher CD8+ TEMRA frequencies were seen in CMV seropositive persons. This confirms that a latent CMV infection could expand CMV-specific T cells to effector or memory cells, which are mostly TEMRA cells [25]. In line with this notion is that the majority of aged CD28− T cells display an EM or TEMRA phenotype [21, 26].

B cells have a key role in allograft outcomes by participating in alloantigen presentation, cytokine production and alloantibody production [27]. Although many studies about the effect of transplantation and immunosuppressive treatments on T cell subset composition and function have been performed, the effects on B cell compartments are still not well known. Inconsistent results have been shown probably caused by differences in immunosuppressive treatment [28–31]. In the present study, difference in B cell subsets frequencies was mainly seen when comparing patients pre- and post-Tx. In line with our results, Llinàs-Mallol et al. showed a higher percentage of memory B cells and lower percentages of naïve and transitional B cells in patients under steroid maintenance immunosuppression 2 yrs after kidney transplantation compared to HC [29].

The effect of ageing on B cells is also controversial. One study showed that the percentage of B cells decreased with age [32] while Qin et al. reported it to be stable in ageing healthy individuals [33]. Our present investigation reveals a more differentiated B cell pool after transplantation, with a marked decreased percentage of naïve and transitional B cells and increased percentage of memory B cells, and especially ABCs after transplantation. ABCs are characterized by the expression of CD21^low^, CD11c+, T-bet+ and CD95^high^, have a low capacity of proliferation, impaired antibody production and reduced telomerase activity. The percentage of peripheral ABCs may increase with age and is elevated in patients with autoimmune diseases such as systemic lupus erythematosus and rheumatoid arthritis [34]. Frasca et al. suggested that ABCs may accumulate as a result of challenges by auto-antigens and chronic exposure to antigens such as CMV since they found higher numbers of ABCs in CMV seropositive individuals compared to CMV seronegative controls [34]. Our results are in line with this theory as we found a significant increase in ABC frequencies after transplantation in patients with viral or bacterial infections during follow-up. No correlation was found between percentages of ABCs and age. However, CMV seronegative patients did show a significant increase of ABC frequencies after transplantation. This could be due to the fact that frequencies of ABCs in CMV seronegative patients were at relative low level before transplantation and the changes after transplantation were more sensitive. Since differences in frequencies were found mainly after transplantation, we speculate that transplantation and immunosuppressive treatment might accelerate senescence of B cells, especially in elderly patients.

This study has several limitations. First, due to the lack of the absolute counts of leukocytes and lymphocytes in fresh blood before peripheral blood mononuclear cells (PBMCs) isolation, the absolute numbers of T and B lymphocyte subsets are not known. However, there were no abnormal lymphocyte counts in the transplant patients during routine lab examinations. Secondly, the numbers of transplant patients and HC were relatively small in this study and all the participants were younger than 70 years. By dividing participants further into subgroups according to their age and CMV serostatus, the numbers are even smaller and this limits the power of comparative testing. Since the age of patients undergoing kidney transplantation is increasing and the prevalence of CMV rises with age, older patients (> 70 years old) should be included in future studies [2, 35]. On the other hand, there was a long follow up of transplant patients and patients were in a stable condition. In this way, we were able to show significant alterations in the composition of B and T cell subsets. A statistical challenge was the multitude of comparisons made between patients and controls, and between patients pre-and post-transplantation (paired data), which made correcting for multiple testing problematic. Because of the very low *p*-values found for the relevant subsets, lack of correction does not impact on the conclusions made. In our study, no relation between frequencies of these subsets and rejection or different immunosuppression regimes (tacrolimus/cyclosporine) was seen (only 6 of 36 patients experienced rejection after transplantation during follow-up). There were no significant differences in B and T cell subsets between patients before transplantation and HC, suggesting that lymphocyte composition was not already altered by the underlying disease that led to kidney transplantation.

## Conclusions

We found an increase in lymphocyte senescence after kidney transplantation reflected by increased frequencies of terminally differentiated T and B cells, and decreased expression of CD28 in both CD4+ and CD8+ T cell populations compared with HC, and this last observation was associated with CMV seropositivity. Further studies are needed to get a better understanding of the ageing molecular mechanisms in the immune system of transplant patients to develop better strategies for improving transplant outcomes.

## Methods

### Study population

Patients who had received a renal transplant in the University Medical Center Groningen between 2 to 3 years earlier were included in the study. At out-patient clinic visits, blood was drawn for collection of PBMCs and serum. PBMCs and serum samples before transplantation were retrieved from diagnostic archives. They were collected before administering induction therapy, immediately prior to the transplantation. HC were age and sex-matched to patients. For transplant patients, the age at transplantation was used as reference point.

Immediately before and 4 days after renal transplantation standard induction therapy using basiliximab (2 doses of 20 mg) was administered. Immunosuppression after transplantation consisted of tacrolimus or cyclosporine, in combination with mycophenolate mofetil and prednisolone. Tacrolimus was switched to cyclosporine if side effects happened. Regarding the dose, we started tacrolimus 0.15–0.2 mg/kg body weight (divided in two doses daily) and cyclosporine was given on dose 5–8 mg/kg body weight (divided in two doses daily). Through levels for cyclosporine were aimed 200–250 mg in the first 3 months and slowly tapered to 75–100 μg/L at the end of the first year. As for tacrolimus, maintenance level was 4–5 mg/kg body weight after 1 year. Prednisolone dose was gradually tapered until a fixed dose of 5 mg was reached. Anti-rejection therapies in 6 of 36 included patients generally consisted of intravenous prednisolone (1000 mg intravenously on three consecutive days, possibly repeated). If patients had infections, immunosuppressant was decreased or stopped temporarily based on seriousness of illness.

Characteristics and medical history of patients were retrieved from medical records. Furthermore, medical records were reviewed for occurrence of acute infections in the period between transplantation and study inclusion (2–3 years after transplantation). Seropositivity for herpes viruses, hepatitis viruses or BK virus was not regarded as an acute infection. We monitored CMV-load of patients after transplantation and (val) ganciclovir was given when deemed clinically necessary. Treatment was stopped when CMV-load < 100 copies/ml for two times.

### Isolation, storage and thawing of PBMCs and serum

Immediately after collection of venous blood in lithium heparin containing tubes, PBMCs were isolated by density gradient centrifugation using lymphoprep (Alere Technologies Inc) according to standard protocols and stored in liquid nitrogen until use. Upon thawing, cell viability was evaluated by trypan blue staining, and was between 85 and 100%. Serum was stored at − 20 °C until use.

### Flow cytometry

For analysis of T cell subsets PBMCs (1.0 × 10^6^ cells/100 μl) were stained with anti-CD3 (Biolegend; 317,344), anti-CD4 (BD biosciences, 345,769), anti-CD8 (BD biosciences, 345,772), anti-CD25 (Biolegend, 356,128), anti-CD127 (BD biosciences, 742,547), anti-CXCR5 (BD biosciences, 564,624), anti-CD45RA (BD biosciences, 562,886), anti-CCR7 (BD biosciences, 557,648), anti-PD1 (BD biosciences,565,299) and anti-CD28 (Biolegend, 302,948). The cells were incubated with the antibody mix for 60 min, protected from light. T cell subsets (CD3+) were defined as follows within CD4+ or CD8+ compartments: naïve cells, CCR7+/CD45RA+; central memory (CM), CCR7+/CD45RA−; effector memory (EM), CCR7−/CD45RA−; effector memory T cells re-expressing CD45RA (TEMRA), CCR7−/CD45RA+; regulatory T cell (Treg), CD4+/CD25+/CD127Low/−; follicular T helper cells (Tfh), CD25 − CD127low/−/CXCR5+/PD1high/+ (within CD4+ CXCR5+); senescent T cells, CD28 − .

For analysis of B cell subsets, PBMCs were stained with anti-CD27 (Biolegend, 356,418), anti-CD11c (Biolegend, 337,206), anti-IgD (BD biosciences, 348,228), anti-IgM (Biolegend, 314,532), anti-CD21 (BD biosciences, 564,437), anti-CD38 (Biolegend, 356,616) and anti-CD19 (BD biosciences, 563,325). The cells were incubated with the antibody mix for 60 min, protected from light. B cell subsets were defined as follows within the CD19+ compartment: naïve cells, CD27−/CD38−; transitional cells, CD27−/CD38+; plasma blast/plasma cells, CD27+/CD38+; memory cells, CD27+/CD38−; switched, IgM−/IgD−; non-switched, IgM+/IgD+; IgM only, IgM+/IgD−; ABCs, CD21−/CD11c + .

Stained PBMCs were measured using the LSR II flow cytometer (BD). The data was processed with the flow cytometry analysis software Kaluza (Beckman Coulter).

### Statistical analyses

Since most of the data were not normally distributed, we performed nonparametric tests in our study. Wilcoxon signed rank test, intended for paired analyses, was used to compare continuous variables before and after transplantation. Results and characteristics of subgroups of patients were compared using a Mann-Whitney test or Wilcoxon signed-rank test when appropriate. For correlations, Spearman’s rho was used. Two-tailed *p*-values ≤0.05 were considered as statistically significant. Statistical analysis was performed using Prism 7 (GraphPad Software, USA).

## Supplementary Information

**Additional file 1.** Supplementary material

## Data Availability

The datasets used and/or analysed during the current study are available from the corresponding author on reasonable request.

## Abbreviations

ABCs: age associated B cells
HC: healthy controls
ESRD: end-stage renal disease
Tx: transplantation
CMV: Cytomegalovirus
MPGN: membranoproliferative glomerulonephritis
FSGS: focal segmental glomerulosclerosis
GBM: glomerular basement membrane
SLE: systemic lupus erythematosus
TIN: tubulointerstitial nephritis
Tac: tacrolimus
Cyc: cyclosporine
MMF: mycophenolate mofetil
Pred: prednisolon
RRT: renal replacement therapy
HD: haemodialysis
PD: peritoneal dialysis
IQR: interquartile range
CM: central memory
EM: effector memory
TEMRA: effector memory T cells re-expresses CD45RA
Tfh: T follicular helper cells
Treg: regulatory T-cell
PB/PC: plasma blast/plasma cell
