# Supplementary material for: Changes in T and B cell subsets in end stage renal disease patients before and after kidney transplantation
Source: Immun Ageing. 2021 Nov 8;18:43. doi: 10.1186/s12979-021-00254-9 (PMC8574047; doi:10.1186/s12979-021-00254-9)
Supplement: Supplementary file 1 — Additional file 1. Supplementary material [file 12979_2021_254_MOESM1_ESM.docx]

Supplementary Material

**
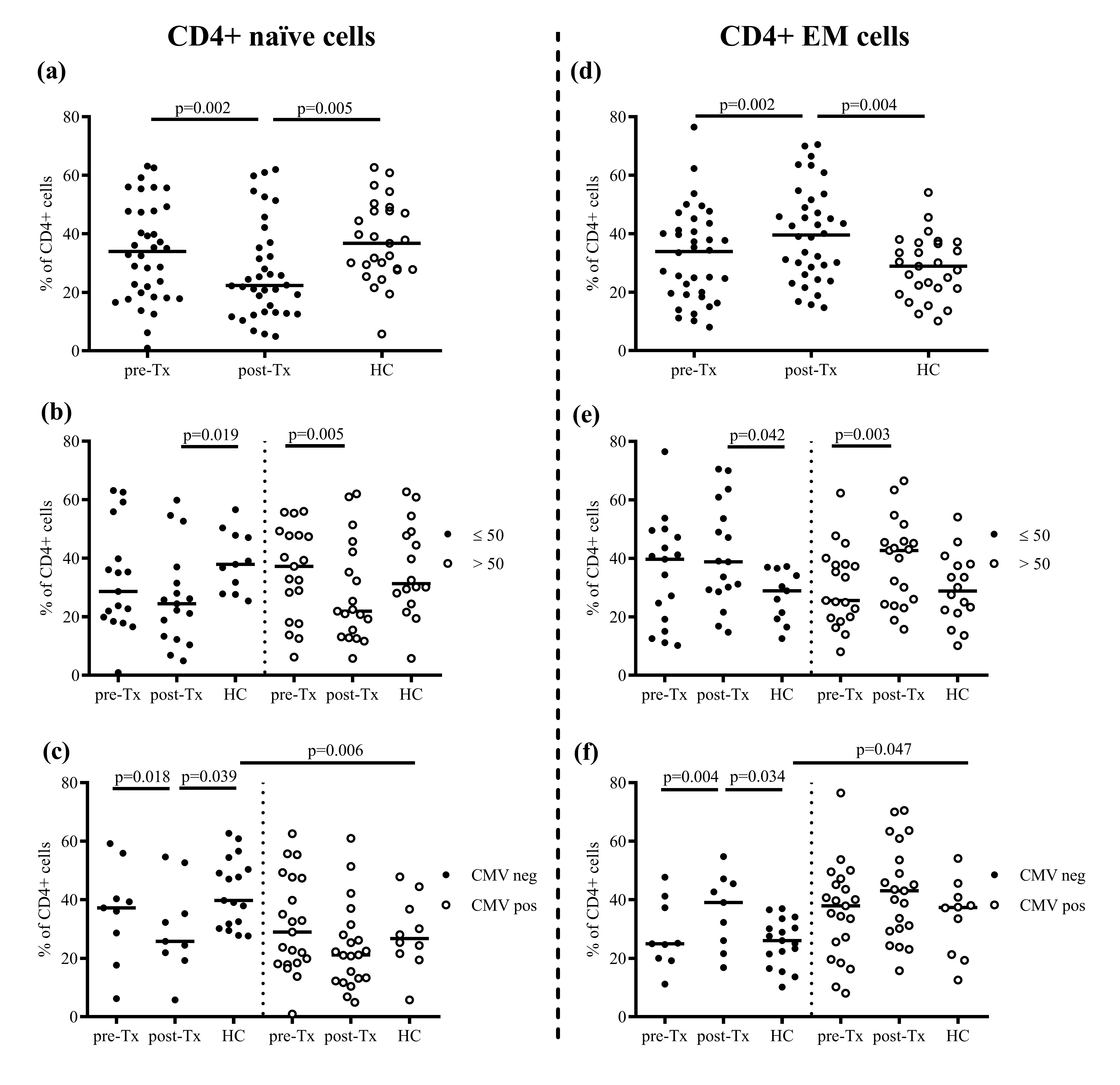
Supplementary Figure 1.** Frequency of CD4+ naïve cells and CD4+ effector memory (EM) cells. Frequency of CD4+ naïve cells within CD4+ cells in all subjects (**a**) and subgroups by their age (≤50 or >50 years old) (**b**) or CMV serostatus (**c**). Frequency of CD4+ EM cells within CD4+ cells in all subjects (**d**) and subgroups by their age (≤50 or >50 years old) (**e**) or CMV serostatus (**f**). Lines show the median. Patients with converted CMV serostatus during study period were not included in (**c**) and (**f**). HC, healthy controls; Tx, transplantation.

**
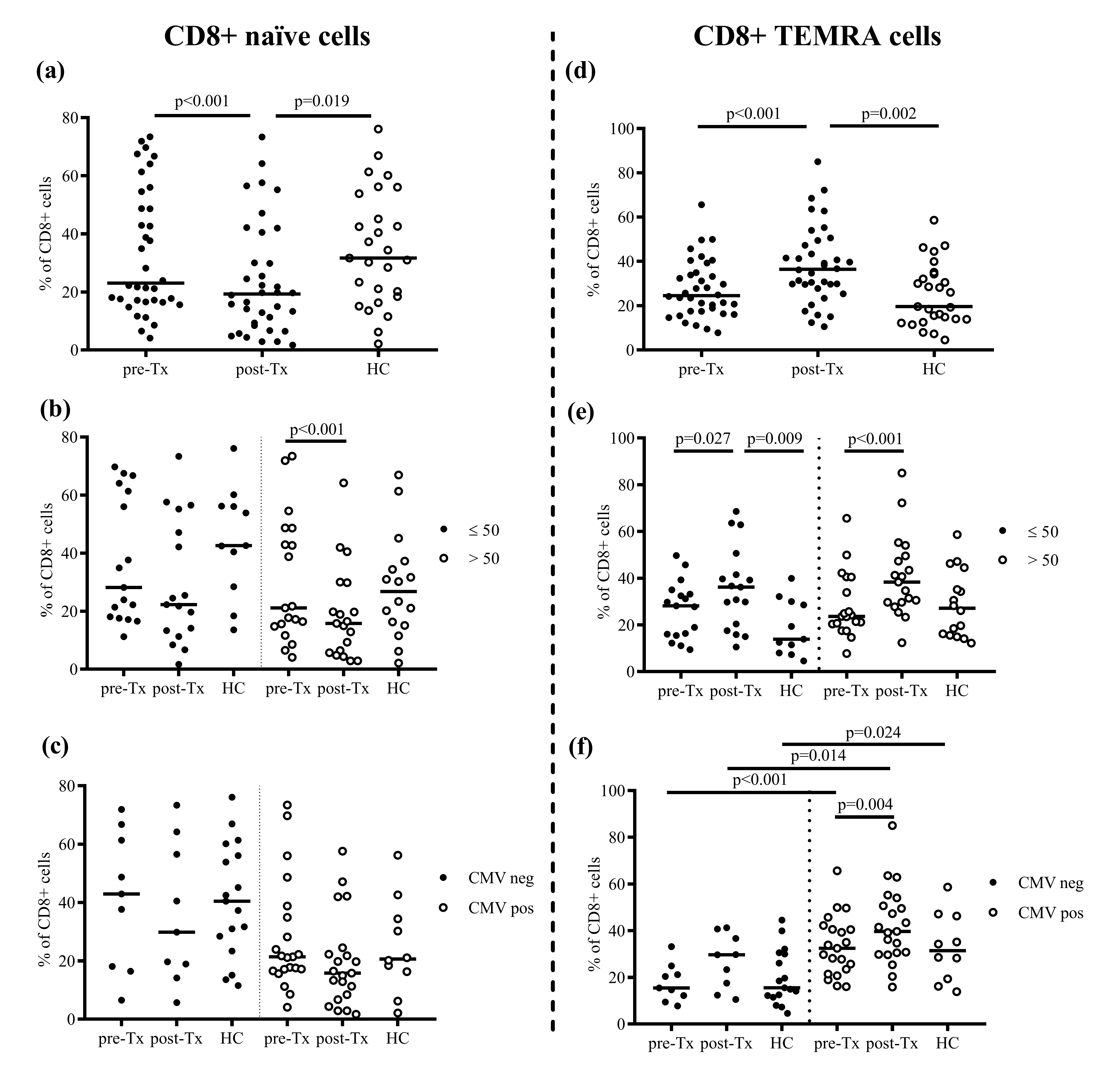
Supplementary Figure 2.** Frequency of CD8+ naïve cells and CD8+ TEMRA cells. Frequency of CD8+ naïve cells within CD8+ cells in all subjects (**a**) and subgroups by their age (≤50 or >50 years old) (**b**) or CMV serostatus (**c**). Frequency of CD8+ TEMRA cells within CD8+ cells in all subjects (**d**) and subgroups by their age (≤50 or >50 years old) (**e**) or CMV serostatus (**f**). Lines show the median. Patients with converted CMV serostatus during study period were not included in (**c**) and (**f**). HC, healthy controls; Tx, transplantation; TEMRA, effector memory T cells re-expresses CD45RA.

**
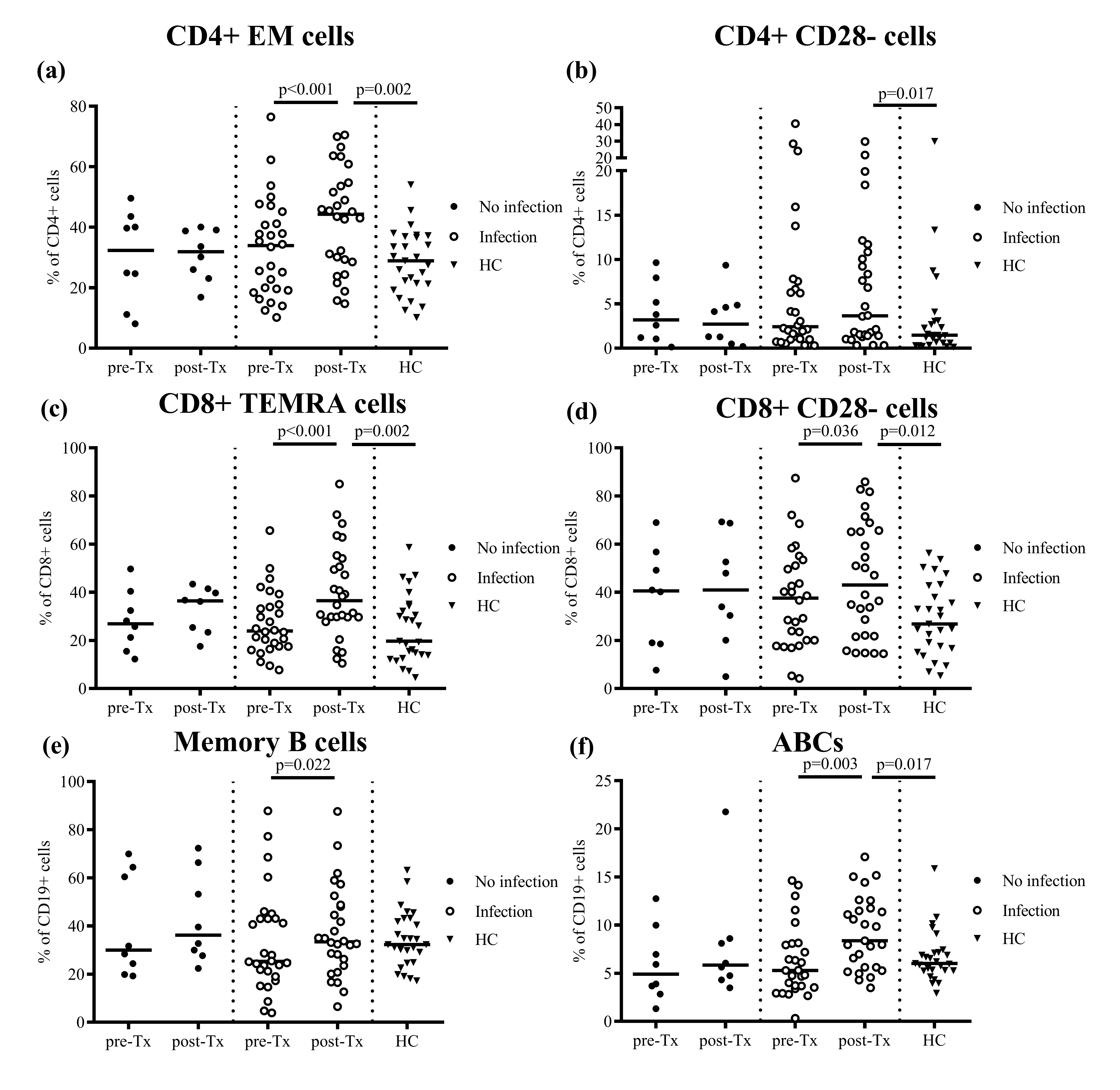
**

**Supplementary Figure 3.** Frequency of differentiated T and B cells. CD4+ EM cells (**a**) and CD4+CD28− cells (**b**) within CD4+ cells; CD8+ TEMRA cells (**c**) and CD8+CD28− cells (**d**) within CD8+ cells; memory B cells (**e**) and ABCs (**f**) within CD19+. Patients were subgroups by their infection situation post-Tx. Lines show the median. HC, healthy controls; Tx, transplantation; EM, effector memory; TEMRA, effector memory T cells re-expresses CD45RA; ABCs, age associated B cells.
